# Climate Change is Rewriting the Hydroclimatic Operating Envelope of Mines

**DOI:** 10.1002/advs.78221

**Published:** 2026-10-14

**Authors:** Haoxuan Yu, Izni Zahidi, Chow Ming Fai, Jilong Pan

**Affiliations:** ^1^ Department of Civil Engineering School of Engineering Monash University Malaysia Bandar Sunway Selangor Malaysia; ^2^ Monash Climate‐Resilient Infrastructure Research Hub (M‐CRInfra) School of Engineering Monash University Malaysia Bandar Sunway Selangor Malaysia; ^3^ United Nations System United Nations University Global Health (UNU Global Health) Cheras Malaysia; ^4^ School of Clinical Medicine Tsinghua University Beijing China; ^5^ School of Resources and Safety Engineering Central South University Changsha China

**Keywords:** climate adaptation, climate safety and health, groundwater, hydroclimatic non‐stationarity, mine closure, mining systems, tailings

## Abstract

Physical climate risk in mining is framed as floods, droughts, heatwaves, and wildfires acting on assets with fixed design conditions. We argue that the deeper change is a moving hydroclimatic operating envelope: climate alters the water and heat conditions around mines, while geology, engineering, and lifecycle stage govern how those changes propagate. Linking a global mine inventory to observed and projected Köppen–Geiger classes shows that 16.2% of 1093 geocoded mines changed climate subtype between 1901–1930 and 1991–2020. By 2071–2099, the share rises to 29.8% under Shared Socioeconomic Pathway 2–4.5 (SSP2–4.5) and 42.5% under Shared Socioeconomic Pathway 5–8.5 (SSP5–8.5). Water trajectories also separate. Under SSP5–8.5, median precipitation falls by 3.1% at arid mines but rises by 6.5% at humid locations, while precipitation seasonality increases in arid regions. Commodity geography stratifies this exposure, whereas mine method alone provides little adjusted separation. Tailings records place weather within multi‐causal failure pathways: 64 of 229 failures with a reported cause included a weather hazard, yet raise categories did not distinguish reported climate‐assessment responses. Literature links these shifts to pore pressure, infiltration, heat, material evolution, water storage, and post‐closure hydrology. The combined evidence supports adaptation based on monitored, scenario‐conditioned operating envelopes rather than static hazard checklists.

## Graphical abstract

Climate change is shown to shift the hydroclimatic operating envelope of mines: 16.2% of represented mine locations have already changed Köppen subtype, rising to 42.5% by late century under SSP5‐8.5. Divergent water trajectories are filtered through geology, infrastructure and lifecycle stage, indicating that adaptation requires monitored, scenario‐conditioned operating envelopes rather than static hazard checklists.

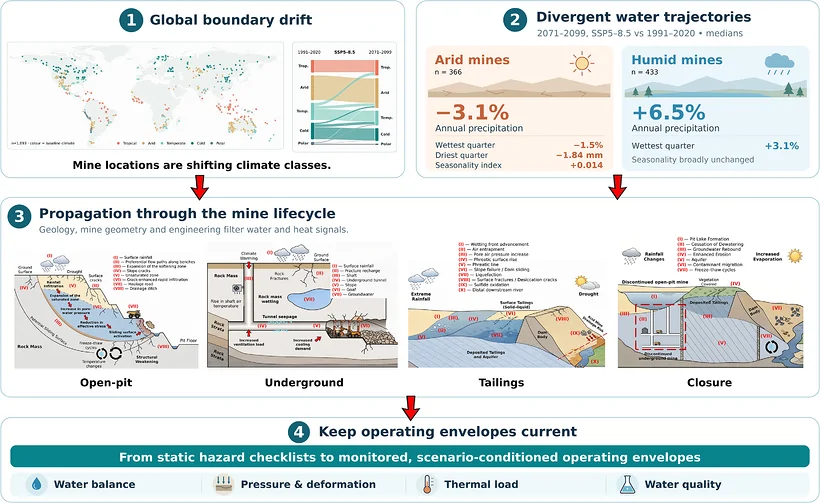

## 1 Climate Risk Begins With a Moving Baseline

The energy transition places mining in a double bind. Mineral supply must expand to support electrification and low‐carbon infrastructure [1], while climate change is already altering infrastructure and industrial systems [2]. Global copper, lead–zinc, and nickel resources are exposed to water scarcity and climate change [3], and mining water security extends beyond operational supply to communities, ecosystems, and institutions [4]. Long‐lived mine facilities therefore carry climatic assumptions far beyond the period in which they were designed [5].

Most climate‐risk assessments begin with discrete hazards, but every hazard acts on a baseline that is itself moving. Historical hydrological stationarity can no longer be assumed [6], and compound impacts often emerge from antecedent conditions, interacting variables, and event sequences [7, 8]. Recharge, groundwater storage, and delayed aquifer responses can transmit climatic change below ground [9, 10]. A design rainfall, phreatic surface, ventilation load, or closure water balance may therefore drift out of alignment with the environment even when no single event is unprecedented.

Our hypothesis is that climate change alters mining risk by shifting water–heat boundary conditions, while baseline hydroclimate, geology, mine geometry, infrastructure, and lifecycle stage govern how that forcing is transmitted. We examine this sequence by linking the Fineprint mine database [11, 12] to observed and projected climate fields from Beck and colleagues [13, 14], including precipitation seasonality described by the Walsh–Lawler index [15]. Independent tailings disclosures and failure records extend the evidence from mine locations to engineered storage systems [16, 17, 18]. Together, these sources reveal where boundary drift occurs and where hydroclimatic responses diverge; published mechanics trace those changes through open pits, underground workings, tailings, and closure. The frozen analytical record, mine‐inventory construction, climate matching, and derivation of hydroclimate variables are detailed in the accompanying Section S1 (Methods S1–S4).

## 2 Mine Climates are Already Crossing Familiar Boundaries

The hydroclimatic operating envelope is already shifting around mines. The 1093 geocoded Fineprint mine locations span all five major Köppen–Geiger groups (Figure 1a), but their commodity and mine‐method composition is strongly structured (Figure 1b) [11, 12, 13, 14]. The represented mine systems therefore enter climate change from fundamentally different water–energy regimes and engineering configurations. Mine‐inventory construction and recorded mining‐method classification are described in Method S2; detailed sample counts and hydroclimate coverage are reported in Results S1.

**FIGURE 1:**
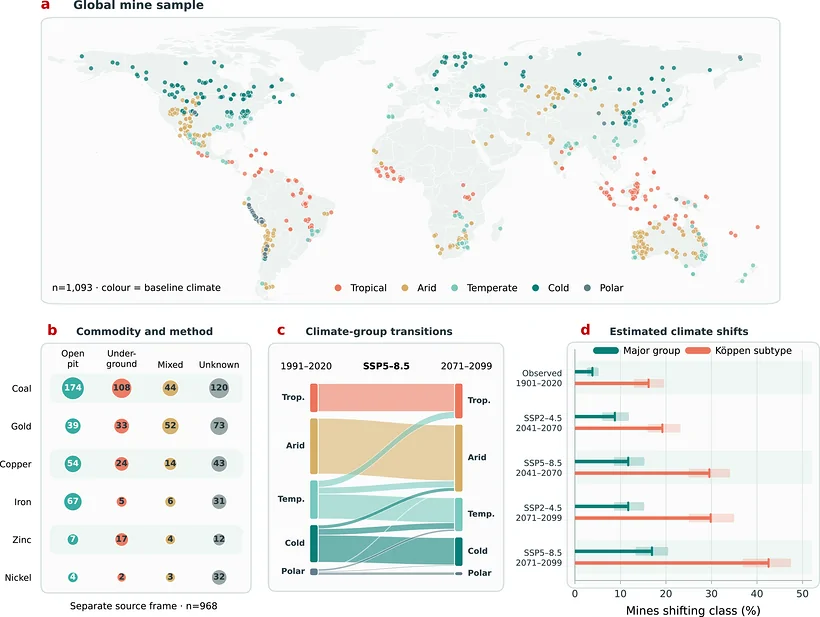
Mine locations in a global public database span shifting hydroclimate boundaries. (a) Locations of 1093 geocoded top‐level mines from the Fineprint database, coloured by 1991–2020 major Köppen–Geiger group. Seventy‐eight of 1171 mine records lacked usable coordinates. (b) Counts of the six most represented commodity groups by harmonized mine method; “Unknown” is retained rather than imputed. (c) Sankey diagram showing transitions between 1991–2020 and 2071–2099 SSP5–8.5 major climate groups. (d) Mine‐location shares changing Köppen subtype and major climate group for the historical‐normal comparison and four future scenario–horizon combinations. Points are estimates and bars are 95% intervals from 1000 one‐degree spatial‐block bootstrap replicates (1093 mines in 627 blocks). These intervals quantify spatial resampling within the linked public‐database sample; they do not incorporate climate‐product, climate‐model, or scenario uncertainty. Data: Fineprint v1.0.3 [11, 12] and Beck et al. [13, 14].

Those baselines have already moved. Between 1901–1930 and 1991–2020, 16.2% of the represented mine locations changed Köppen subtype (95% interval, 12.95%–19.55%). By late century under Shared Socioeconomic Pathway 5–8.5 (SSP5–8.5), major‐group transitions are multidirectional rather than a uniform poleward relabelling (Figure 1c). Relative to 1991–2020, the projected subtype‐shift share reaches 29.8% under Shared Socioeconomic Pathway 2–4.5 (SSP2–4.5) and 42.5% under SSP5–8.5 by 2071–2099 (Figure 1d). Major climate groups move less often, but the rising subtype signal shows that design environments can change substantially within familiar regional labels. Climate‐group definitions, boundary‐shift indicators, and spatial‐block bootstrap procedures are described in Methods S5 and S6; additional transition estimates and scenario contrasts are reported in Results S2.

These class changes matter because they represent changing water and heat conditions whose engineering consequences depend on the baseline hydroclimate.

## 3 Divergent Water Trajectories Reshape Open Pit Boundary Conditions

The engineering significance of boundary movement depends first on its direction. Under late‐century SSP5–8.5, median annual precipitation falls by 3.1% at arid mine locations but rises by 6.5% at humid locations (Figure 2a). Wettest‐quarter precipitation follows the same contrast, decreasing by 1.5% in arid settings and increasing by 3.1% in humid settings (Figure 2b). The arid driest quarter loses a further 1.84 mm (Figure 2c), while its seasonality index rises by 0.014; the humid index remains broadly unchanged (Figure 2d). The same global forcing therefore produces opposing water‐balance trajectories. Hydroclimate‐variable construction and baseline‐regime definitions are provided in Methods S4 and S5, with detailed wet–arid contrasts in Results S2.

**FIGURE 2:**
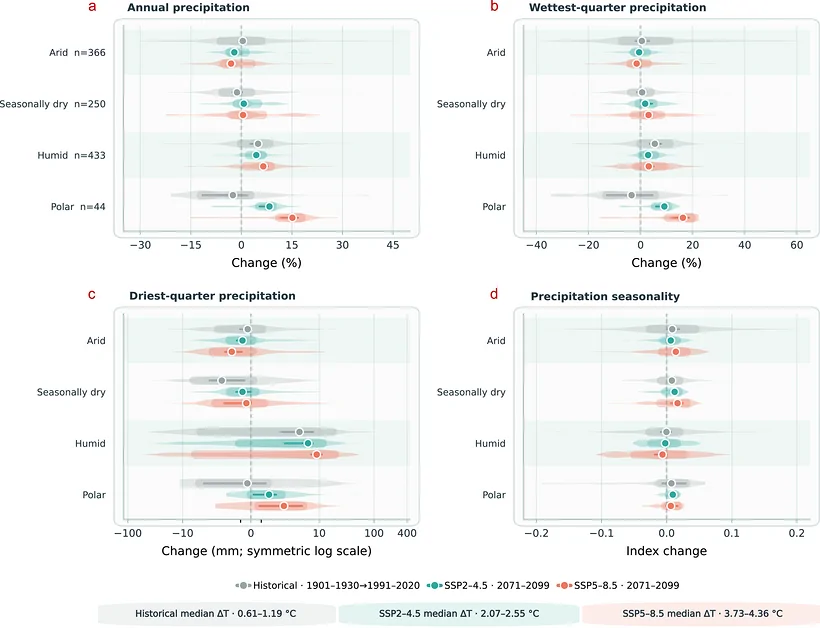
Mine‐region hydroclimate trajectories diverge by baseline regime. Shaded distributions summarize mine‐level changes, and overlaid points and horizontal intervals show medians with 95% one‐degree spatial‐block bootstrap intervals for the historical change (1901–1930 to 1991–2020) and late‐century changes (1991–2020 to 2071–2099 under SSP2–4.5 and SSP5–8.5). Baseline regime is defined from the 1991–2020 Köppen subtype. (a) Percentage change in twelve‐month precipitation. (b) Percentage change in the maximum circular consecutive three‐month precipitation total. (c) Absolute change in the minimum circular consecutive three‐month precipitation total; the panel uses a symmetric‐logarithmic axis with a 10 mm linear region. (d) Change in the Walsh–Lawler precipitation seasonality index. Sample sizes are arid, 366; seasonally dry, 250; humid, 433; and polar, 44 mines. Intervals quantify spatial resampling within the linked sample and exclude climate‐product, model, and scenario uncertainty. Monthly normals describe background conditions and do not resolve storm extremes. Data and climate‐product documentation: Beck et al. [13, 14]; precipitation‐seasonality metric: Walsh and Lawler [15].

These trajectories create different engineering pressures. In humid settings, rising annual and wet‐quarter precipitation can increase conveyance, storage, erosion, and seepage demands. In arid settings, declining annual supply can coexist with stronger precipitation concentration, coupling water scarcity [3, 4] with shorter, wetter periods. Annual precipitation alone would miss both pathways.

On an open‐pit slope, the two pathways converge on effective stress. Terzaghi's relation links total normal stress σ_*n*_, pore‐water pressure *u*, and effective normal stress σ′ [19]:

(1) $$\sigma^{\prime }=\sigma_{n}\;-u$$

and the corresponding Mohr–Coulomb shear strength is

(2) $$\tau_{f}=c^{\prime }+\left(\sigma_{n}-u\right)\tan\varphi^{\prime }$$

As *u* rises, available shear strength falls even if external loading remains unchanged. Wetting and drying can also alter stiffness and damage in sandstone and clay‐bearing rocks [20, 21, 22]. Desiccation cracks redirect infiltration [23], while weak‐rock slope response depends on the interaction between material properties and hydrological conditions [24]. The broader landslide literature shows that rainfall thresholds vary with antecedent water and regional geology [25, 26].

Wetter regimes increase the potential for runoff, infiltration, and transient pore‐pressure rise, whereas arid regimes can combine drying cracks with short, concentrated wet periods. Thermal cycling and freeze–thaw add further changes to discontinuities and frozen rock [27, 28]. Geological and operational filters govern how strongly these signals reach a mine (Figure 3).

**FIGURE 3:**
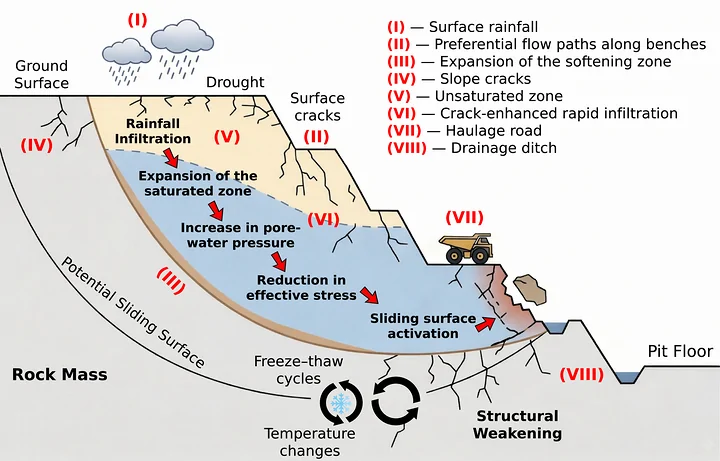
Literature‐based mechanism linking climate boundary shifts to open‐pit slope response. Rainfall and recharge can alter pore‐water pressure and effective stress, while drought cracking, wetting–drying, thermal cycling, and freeze–thaw can modify infiltration pathways and material properties [19, 20, 21, 22, 23, 24, 25, 26, 27, 28].

## 4 Geology Filters the Climate Signal Underground

Surface forcing reaches mine systems through geology and geometry, and the commodity map makes that filtering visible. Coal locations are predominantly humid, whereas gold and copper locations are more strongly represented in arid settings (Figure 4a). All six focal commodity groups warm by about 4°C under late‐century SSP5–8.5, yet their precipitation trajectories and subtype‐shift shares differ markedly (Figure 4b) [11, 12, 13, 14]. Commodity therefore organizes exposure through mine geography, but it does not measure host rock or hydrostratigraphy.

**FIGURE 4:**
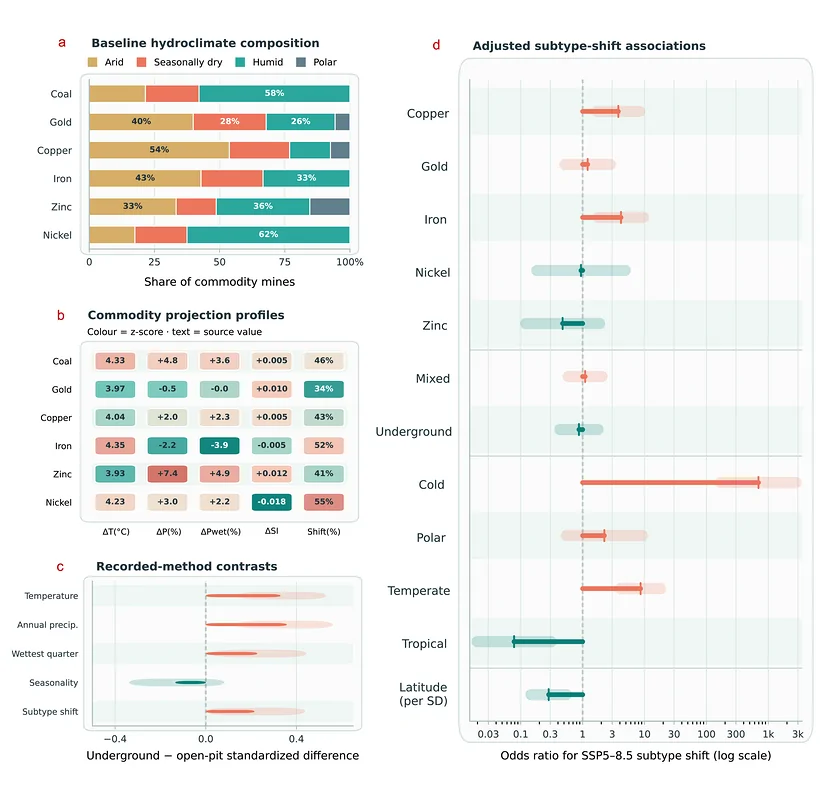
Climate‐boundary exposure differs across commodity and recorded mine method. (a) Baseline hydroclimate composition of coal, gold, copper, iron, zinc, and nickel mines; in‐bar labels show selected within‐commodity percentages. (b) Commodity‐specific medians for late‐century SSP5–8.5 temperature, annual precipitation, wettest‐quarter precipitation, and seasonality changes, plus the share changing Köppen subtype. Cell colour is a within‐metric standardized value; annotations give the physical value. Temperature is the January–November common‐month mean. (c) Unadjusted standardized difference between underground and open‐pit locations for five projected metrics. Positive values indicate larger values at underground‐mine locations. Points and bars are estimates and 95% one‐degree spatial‐block bootstrap intervals (379 open‐pit and 209 underground mines; 369 blocks). (d) Adjusted odds ratios from a logistic model of late‐century SSP5–8.5 subtype shift among 633 mines in 374 blocks. The model includes commodity, method, 1991–2020 major climate group, and standardized absolute latitude; covariance is clustered by one‐degree block. Commodity is a location‐level stratifier, not a proxy measurement of orebody geology. Data: Fineprint and Beck et al. [11, 12, 13, 14].

Recorded mine method shows a similar distinction between category and mechanism. Underground locations have larger unadjusted changes in temperature, annual precipitation, and wettest‐quarter precipitation than open‐pit locations (Figure 4c). In the separate adjusted model of subtype movement, which includes commodity, baseline climate and latitude, the underground‐to‐open‐pit odds ratio is 0.87 (95% confidence interval, 0.43–1.77). In contrast, copper and iron locations retain higher odds than coal locations, with adjusted odds ratios of 3.77 and 4.16, respectively, and Benjamini–Hochberg adjusted values of 0.0038 [29] (Figure 4d). The residual commodity signal may therefore contain geological and regional structure that a method label cannot resolve. Full mine‐level model specifications and complementary method contrasts are detailed in Method S7, with missing‐data and multiplicity rules in Method S10; additional commodity‐ and method‐stratified estimates are reported in Results S3.

Underground response depends on the processes hidden by those labels. Mining‐induced fractures can connect rainfall to shallow coal workings [30], while a 1010 m‐deep coal mine illustrates how depth, thermodynamic conditions, and cooling control shape heat hazards [31]. Water‐sensitive clay‐bearing and argillaceous rocks add material‐specific responses [21, 22]. The same surface signal can therefore arrive rapidly through connected fractures, be delayed by low‐permeability strata, or bypass workings altogether.

Depth, connectivity, hydrostratigraphy, and dewatering can shape mine inflow and pressure response [9, 10, 30], whereas depth, ventilation, and thermal conditions govern temperature and cooling demand in deep workings [31]. Geological control is therefore expressed through measurable flow, strength, and thermal properties, not through commodity name alone (Figure 5).

**FIGURE 5:**
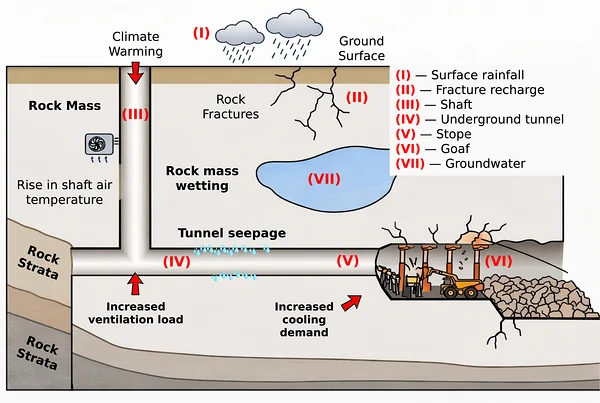
Literature‐based mechanism linking surface climate to underground mine response. Hydrostratigraphy, mining‐induced fractures, depth, dewatering, ventilation, and thermal conditions filter and delay water and heat signals. Clay‐bearing and argillaceous materials show water‐sensitive mechanical pathways [21, 22], desiccation cracks create preferential infiltration pathways [23], and shallow and deep coal mines illustrate rainfall‐connectivity and thermal‐control pathways, respectively [30, 31].

## 5 Tailings Expose Climate Design and Governance as One System

Tailings storage makes geological and engineering filtering especially visible because climatic loading acts through a constructed, water‐retaining system. Two complementary datasets trace this pathway from reported assessment practice to failure triggers and consequences (Figure 6a) [16, 17, 18]. Because facility disclosures and historical failure records describe different populations, they do not form a common denominator for a failure rate. Facility harmonization and the disclosure model are detailed in Method S8, and failure‐event coding and conditional consequence analyses in Method S9; extended results are reported in Results S4.

**FIGURE 6:**
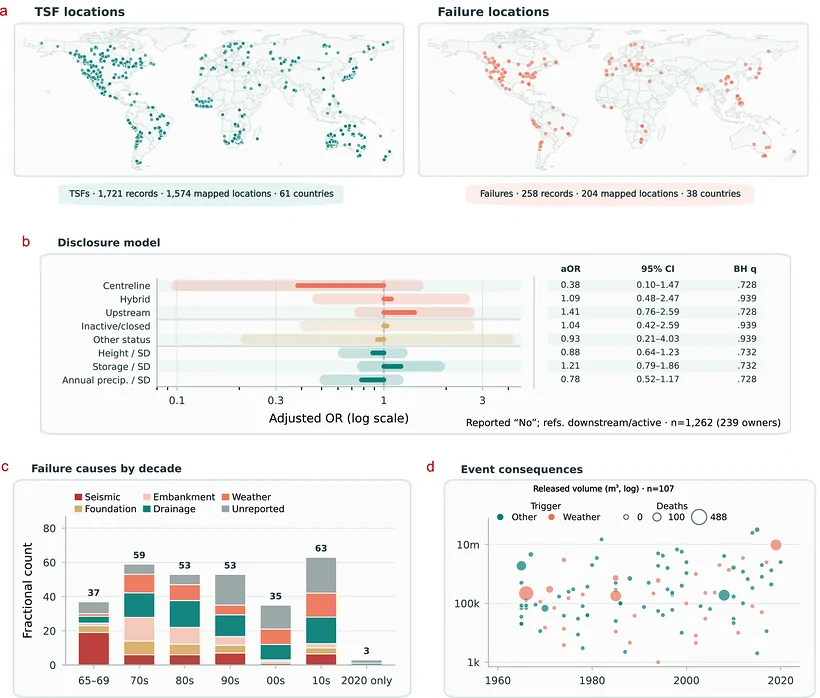
Tailings evidence separates assessment, reported triggers, and consequences. (a) Facilities with usable numeric coordinate pairs in the Franks disclosure dataset (1721 of 1743) and failures with usable numeric coordinate pairs in the Rana repository version (258 of 303). These are complementary datasets, not a common denominator. (b) Adjusted odds ratios for a reported “No” to the question of whether a climate/extreme‐weather assessment had been completed or was planned, among 1262 facilities in 239 owner clusters. Bars are cluster‐robust 95% intervals; the model includes raise category, status, standardized log height, standardized log storage, and standardized annual precipitation. This disclosure item is not an audited safety endpoint. (c) Fractionally counted failure‐cause labels by decade. Multi‐labelled events contribute a total weight of one; 74 events without a reported cause remain “Unreported.” The 1960s bin covers 1965–1969 and the 2020s bin contains 2020 only. (d) Positive released‐tailings volumes plotted against failure year for 107 events with a known cause. Colours distinguish weather‐involved failures from other known‐cause failures, marker size indicates reported deaths, and release volume is shown on a logarithmic scale. Consequence comparisons are conditional on inclusion as a reported failure. Data: Franks et al. [16] and Rana et al. [17, 18].

The apparent design signal is weak: common raise categories did not provide a precise adjusted ranking of reported climate‐assessment responses (Figure 6b). In the failure records, 64 of 229 events with a reported cause included a weather hazard, while drainage, embankment, foundation, and seismic causes frequently co‐occurred (Figure 6c). Weather is therefore part of a multi‐causal pathway rather than a standalone explanation.

The reported consequences reinforce that interpretation. Eleven of 64 weather‐involved failures were fatal, compared with 16 of 165 failures assigned other known causes. Yet the median reported release volume was 150,000 m^3^ for weather‐involved failures and 195 000 m^3^ for other known causes (Figure 6d and Results S4). Event severity cannot be ordered from weather involvement alone.

The mechanism begins with water balance. Rainfall‐induced groundwater ridging and the Lisse effect can rapidly raise local phreatic surfaces through transient pressure transmission [32]. Whether that rise becomes destabilizing depends on drainage, deposition, construction quality, material state, and surveillance [33, 34]. Alternative tailings approaches redistribute environmental, social, and economic trade‐offs [35], while wetting–drying and freeze–thaw cycles can change the hydraulic conductivity of modified tailings [36].

Climatic loading changes inflow, storage, phreatic response, erosion, and material condition within a facility whose performance also depends on operation and governance. The Global Industry Standard on Tailings Management embeds these dependencies in integrated knowledge, monitoring, and lifecycle oversight [37], while historical failure and environmental reviews show interacting causes and widely varying consequences [38, 39]. Together, these climatic, hydraulic, operational, and governance pathways shape tailings performance (Figure 7), and they continue after production ends.

**FIGURE 7:**
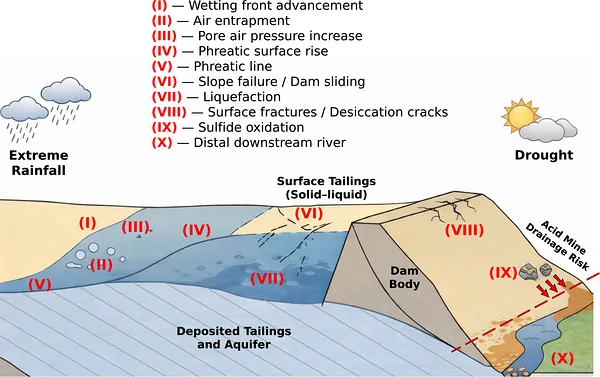
Literature‐based mechanism for climate‐conditioned tailings performance. Rainfall can affect facility water balance and phreatic response [32], while wetting–drying and freeze–thaw can alter material state [36]. Flow‐failure analyses, failure reviews, and the global standard show that drainage, construction, operation, and governance condition whether adverse loading becomes consequential [33, 34, 37, 38, 39]. Alternative tailings approaches involve environmental, social, and economic trade‐offs [35].

## 6 Closure Keeps the Operating Envelope in Motion

Stopping dewatering begins a new hydrological phase rather than ending the old one. Groundwater rebound can unfold over years to decades in open‐pit and underground systems [40, 41, 42]. Rising water then feeds pit‐lake development [43, 44], first‐flush releases from abandoned workings [45], and evolving mixtures of hydrological and geochemical sources [46, 47]. Closure therefore inherits both the mine geometry and the climatic trajectory established during operation.

Climate‐sensitive closure must also account for reconstructed landforms, erosion, and long‐term tailings performance [48, 49]. Government guidance places closure planning early in the mine lifecycle [50], while recent analysis shows that future climate can change closure water balances and management needs [51]. Groundwater rebound, pit lakes, mine‐water flushing, geochemistry, and landform evolution unfold over different response times, yet remain linked to decisions made during operation (Figure 8). Decisions that set today's slopes, dumps, tailings, and drains also shape the system that future owners or the public will inherit.

**FIGURE 8:**
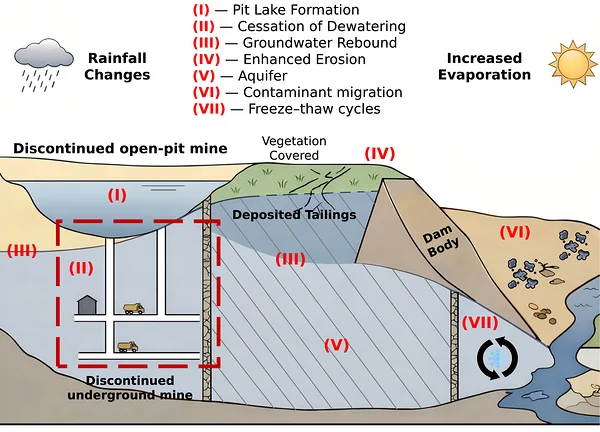
Literature‐based mechanism extending the hydroclimatic operating envelope through closure. Material cycling can continue under freeze–thaw and wetting–drying [27, 36]. Cessation of dewatering, groundwater rebound, pit‐lake development, mine‐water flushing, and geochemical evolution unfold over different lags [40, 41, 42, 43, 44, 45, 46, 47], while reconstructed landforms and closure governance extend the response over longer timescales [48, 49, 50, 51]. The diagram identifies monitoring targets and lifecycle dependencies, not a deterministic sequence.

## 7 Operating Envelopes Must Follow the Mine Lifecycle

Once the mine is viewed across this full timescale, adaptation can no longer be a one‐off design check. A hydroclimatic operating envelope should connect climatic forcing to observable system states, including water balance, pressure and deformation, thermal load, and water quality. Scenario‐conditioned thresholds can then trigger investigation, operational change, or design review before historical assumptions become unsafe.

Keeping that envelope current requires models to exchange boundary conditions across the mine. Pit dewatering changes groundwater [40, 41, 42]; groundwater affects underground inflow [9, 10, 30] and tailings seepage [32, 33, 34]; drainage and storage choices shape downstream water [4, 37]. Non‐stationary precipitation standards provide one example of how design values can be updated without assuming a single certain future [52]. Closure scenarios and financial assurance should evolve with the same evidence rather than enter only when production approaches its end [50, 51].

Because mine water crosses lease and lifecycle boundaries, the operating envelope is also social. Mining legacies propagate through river systems [53], and altered water access or contamination can affect communities and human rights [54]. Portfolio averages can conceal concentrated local exposure, particularly where climate extremes cluster in space and time [55, 56]. Adaptation should therefore track who bears changing withdrawals, releases, and post‐closure obligations as closely as it tracks asset reliability.

## 8 Testing the Operating Envelope Hypothesis

Global screening locates boundary drift, but mine‐scale observations are required to establish how it propagates through hydrogeology, geometry, and engineered systems. Uneven public mine coverage and gridded climate normals bound spatial inference [11, 12, 13, 14], while tailings disclosures and historical failures have distinct sampling frames [16, 17, 18]. The causal test therefore lies at the mine scale. Sensitivity contrasts and software/reproducibility details are documented in Methods S11 and S12, respectively; additional study limitations are summarized in the Supplementary Limitations.

Three observations would narrow the operating‐envelope hypothesis. First, a gridded boundary shift that is not followed by change in recharge, inflow, pressure, temperature, or material state would have little operational value at that site. Second, commodity contrasts that disappear after hydrostratigraphy and mine geometry are measured should not be retained as predictors. Third, climate‐conditioned models that fail to outperform stationary designs on held‐out periods would not justify dynamic updating.

A decisive test would combine global screening with nested site observatories across arid and humid climates, open‐pit and underground mines, contrasting hydrostratigraphies, and operational and closed states. Shared measurements of meteorology, subsurface conditions, deformation, operations, and water quality would allow distributed‐lag and coupled‐process models to predict held‐out periods. The resulting evidence would show when boundary drift becomes an engineering signal and when it remains only a regional climate indicator.

## 9 Conclusion

Climate change is rewriting the background water–heat conditions in which mines are designed, operated, and closed. Global data show that those conditions are moving and that their direction differs across hydroclimates, while the mechanistic evidence shows how geology, infrastructure, and lifecycle stage filter the resulting response. Whether boundary drift becomes instability, interruption, or contamination depends on monitored site processes and engineering choices. Mine adaptation should therefore follow changing operating envelopes across the full lifecycle, linking regional climate trajectories to observations that can trigger timely design and management decisions.

## 10 Quantitative Methods Summary

Mine locations were linked to historical and projected Köppen–Geiger classes and monthly climate normals from the cited public datasets [11, 12, 13, 14]. Spatial‐block bootstrap intervals account for clustering among nearby mine locations, and adjusted models describe commodity, method, and tailings‐disclosure associations without assigning causality. Facility disclosures and reported tailings failures were analysed independently [16, 17, 18]. Full details of data processing, spatial matching, derived hydroclimate metrics, statistical models, sensitivity contrasts, and software are provided in the accompanying Section S1 (Methods S1–S12), together with extended numerical results (Results S1–S4).

## Author Contributions

Conceptualization, H.Y. and I.Z.; methodology, H.Y.; validation, I.Z. and J.P.; formal analysis, H.Y. and J.P.; resources, H.Y.; Writing – original draft preparation, H.Y.; Writing – review and editing, H.Y., I.Z., C.M.F. and J.P.; visualization, H.Y. and J.P.; supervision, I.Z. and C.M.F.; project administration, H.Y. All authors have read and agreed to the published version of the manuscript.

## Conflicts of Interest

The authors declare no conflicts of interest.

## Supporting information




**Supporting File 1**: advs78221‐sup‐0001‐SuppMat.docx.

**Supporting File 2**: advs78221‐sup‐0002‐SuppMat.zip.

## Data Availability

The detailed data that support the findings of this study are available from the corresponding author upon reasonable request. Resulting data are also supplied in the accompanying ZIP archive (Section S2). All source datasets analysed in this study are publicly available. The Fineprint global coal and metal mine database (version 1.0.3) is available from Zenodo under CC BY 4.0 (https://doi.org/10.5281/zenodo.7369478) [12]. The Köppen–Geiger climate‐classification and climate‐normal products are available from Figshare (https://doi.org/10.6084/m9.figshare.c.6395666.v1) [14]. Tailings‐facility disclosure data are provided in the supplementary spreadsheet published with Franks et al. (source‐file title: “Section S2”) (https://doi.org/10.1038/s41598‐021‐84897‐0) [16]. The tailings‐failure database (version 2) is available from Borealis under CC0 (https://doi.org/10.5683/SP3/K7SANT) [18]. Analytical procedures and extended numerical results are provided in the accompanying Section S1.
