# Supplementary material for: Climate Change is Rewriting the Hydroclimatic Operating Envelope of Mines
Source: Adv Sci (Weinh). 2026 Oct 14:e78221. Online ahead of print. doi: 10.1002/advs.78221 (PMC13668327; doi:10.1002/advs.78221)
Supplement: Supplementary file 1 [file ADVS-9999-e78221-s002.docx]

**Supporting Information S1**

**Climate change is rewriting the hydroclimatic operating envelope of mines**

Supporting Information S1. Extended methods, results and limitations (DOCX). This file contains Supplementary Methods S1–S12, Supplementary Results S1–S4, Supplementary Limitations and Supplementary References.

The accompanying resulting-data archive is supplied separately as Supporting Information S2 (ZIP).

## Supplementary Methods

## Supplementary Method S1. Study design and frozen analytical record

The analysis was completed on 4 September 2026 using four openly accessible sources: the Fineprint global coal and metal mine database, the Beck et al. Köppen–Geiger climate-classification and climate-normal collection, the Franks et al. tailings-facility disclosure dataset, and version 2 of the Rana et al. tailings-failure database [1–4]. Fineprint v1.0.3 was obtained from Zenodo (dataset DOI 10.5281/zenodo.7369478; CC BY 4.0), the Beck collection from Figshare (collection DOI 10.6084/m9.figshare.c.6395666.v1), and the Rana database from Borealis (dataset DOI 10.5683/SP3/K7SANT; CC0 1.0). The Franks facility records came from the supplementary spreadsheet published with the cited open-access article [3] (source-file title: “Supplementary Information 2”; XLSX). The random-number seed was fixed at 20260904. All reported spatial-block bootstrap intervals used 1,000 replicates. These intervals quantify spatial resampling within the linked public-database sample and do not include climate-product, climate-model, or scenario uncertainty. No missing value was imputed.

The evidence chain was prespecified at four levels. First, we measured whether the background climate class assigned to represented mine locations changed between historical, current and projected 30-year normals. Second, we compared continuous precipitation and temperature changes among baseline arid, seasonally dry, humid and polar settings. Third, we assessed whether the projected climate-boundary response was stratified by commodity and recorded mining method. Fourth, we examined a separate body of tailings evidence, distinguishing self-reported facility conditions from reported failure events. All results are descriptive associations or scenario contrasts.

## Supplementary Method S2. Mine locations, commodities, mining methods and production weights

We used version 1.0.3 of the Fineprint database [1]. The GeoPackage contained mines and associated subsites. The analytical inventory was restricted to records whose facility type began with “Mine” and whose subsite name was empty. This produced 1,171 top-level mine records in 63 countries. Of these, 1,093 had valid point coordinates.

The primary commodity field was retained as supplied. Six groups with sufficient representation were prespecified for comparative analysis: coal, gold, copper, iron, zinc and nickel. The remaining commodities were retained as “Other” in descriptive summaries but were excluded from the mine-level regression.

Mining method was derived from the free-text facility-equipment field. A record containing “underground” was classified as underground; a record containing “open pit”, “open cut” or “surface” was classified as open pit; a record containing both an underground and a surface term was classified as mixed; all other or missing records were classified as unknown. This reproducible rule yielded 402 open-pit, 221 underground, 142 mixed and 406 unknown records. The method label therefore describes recorded equipment or configuration and is not an independently verified engineering classification.

Production was used only to construct a secondary exposure weight. For 2015–2020, positive “Coal mined” records were selected from the coal table. Positive commodity-output records coded as Me.Au, Me.Cu, Me.Fe, Me.Zn and Me.Ni were selected from the commodities table and mapped to gold, copper, iron, zinc and nickel, respectively. Duplicate facility–year–commodity rows were summed. The weight for mine $i$ was the mean of its available positive annual values:

$w_{i}=\frac{1}{n_{i}}\sum_{t\in\mathcal{T}_{i}}Q_{i,t},$	(S1)

where $Q_{i,t}$ is reported output in tonnes and $\mathcal{T}_{i}$ is the set of available years from 2015 through 2020. Missing years remained missing and were not recoded as zero. A positive weight was available for 658 mines, of which 641 also had climate coordinates. Because physical tonnage is not economically comparable across commodities and coverage is non-random, this quantity is retained only as an exploratory diagnostic. Coal contributes 95.75% of the summed weights, followed by iron at 3.70%; the resulting estimate must not be described as a global production-weighted risk or exposure.

## Supplementary Method S3. Climate classifications, climate normals and spatial matching

We used the Beck et al. high-resolution Köppen–Geiger collection [2]. Köppen–Geiger classes were sampled from the 0.1° GeoTIFF products. Monthly precipitation and air-temperature normals were sampled from the corresponding 0.5° ensemble-mean NetCDF products. The analysed periods were 1901–1930, 1991–2020, 2041–2070 under Shared Socioeconomic Pathway 2–4.5 (SSP2–4.5) and Shared Socioeconomic Pathway 5–8.5 (SSP5–8.5), and 2071–2099 under SSP2–4.5 and SSP5–8.5.

Each geocoded mine was linked to the raster cell containing its coordinate. If a 0.1° Köppen–Geiger cell was unclassified, the nearest classified cell within five 0.1° cell rings was used. This affected one mine, at a displacement of 0.1°. For the 0.5° monthly normals, the nearest grid-cell centre with complete precipitation and January–November temperature values was used within a search radius of three grid cells. The median mine-to-climate-cell-centre distance was 0.196°, the 99th percentile was 0.345°, and the maximum was 0.901°. Nine sites were linked farther than the half-diagonal of a 0.5° grid cell because the nominal cell lacked complete values. These distance diagnostics were inspected before the linked records entered the analysis.

Quality control identified an archive-specific defect: the December air-temperature layer in the 2071–2099 SSP5–8.5 0.5° ensemble-mean file was populated throughout by the invalid value −9999.3125 rather than valid temperatures. All 259,200 cells in that layer failed the prespecified physical range check of −100 to 100 °C. To maintain a like-for-like calendar basis across every historical and scenario comparison, temperature normals were therefore calculated from the common complete January–November months for all periods and are not described as annual means. Precipitation variables use all 12 months.

## Supplementary Method S4. Derived hydroclimate variables

For mine $i$, period $k$ and calendar month $m$, monthly precipitation was denoted $P_{i,k,m}$. Negative precipitation values, if present, were set to zero before aggregation. Annual precipitation was

$P_{\mathrm{ann},i,k}=\sum_{m=1}^{12}P_{i,k,m}$	(S2)

Wettest- and driest-quarter precipitation were calculated from all 12 cyclic, consecutive three-month windows, including windows that cross December and January:

$P_{wet3,i,k}=\max_{s\in\{1,\ldots,12\}}(\sum_{r=0}^{2}P_{i,k,\{(s+r-1)\mathrm{mod} 12\}+1}),$	(S3)

$P_{dry3,i,k}=\min_{s\in\{1,\ldots,12\}}(\sum_{r=0}^{2}P_{i,k,\{(s+r-1)\mathrm{mod} 12\}+1})$	(S4)

Precipitation seasonality was summarized by the Walsh–Lawler index [5]:

${SI}_{i,k}=\frac{1}{P_{\mathrm{ann},i,k}}\sum_{m=1}^{12}\left| P_{i,k,m}-\frac{P_{\mathrm{ann},i,k}}{12} \right|$	(S5)

The temperature metric was

$T_{\mathrm{JN},i,k}=\frac{1}{11}\sum_{m=1}^{11}T_{i,k,m},$	(S6)

where $T_{\mathrm{JN}}$ denotes the January–November mean. For a reference period $r$ and target period $k$, temperature and seasonality changes were simple differences. Annual- and wettest-quarter precipitation changes were expressed as percentages of the reference value. Driest-quarter precipitation was expressed as an absolute millimetre difference because percentage changes become unstable when the reference dry-quarter total approaches zero:

$\Delta T_{i,r\to k}=T_{\mathrm{JN},i,k}-T_{\mathrm{JN},i,r},$	(S7)

$\Delta P_{i,r\to k}(\%)=100\times\frac{P_{\mathrm{ann},i,k}-P_{\mathrm{ann},i,r}}{P_{\mathrm{ann},i,r}},$	(S8)

$\Delta P_{wet3,i,r\to k}(\%)=100\times\frac{P_{wet3,i,k}-P_{wet3,i,r}}{P_{wet3,i,r}},$	(S9)

$\Delta P_{dry3,i,r\to k}=P_{dry3,i,k}-P_{dry3,i,r},$	(S10)

$\Delta{SI}_{i,r\to k}={SI}_{i,k}-{SI}_{i,r}$	(S11)

The historical comparison used 1901–1930 as the reference and 1991–2020 as the target. Scenario comparisons used 1991–2020 as the reference.

## Supplementary Method S5. Climate groups, hydroclimate regimes and boundary shifts

The 30 Köppen–Geiger subtypes were retained for subtype-transition analyses. Major climate groups were defined by the first letter of the Köppen–Geiger code: A, tropical; B, arid; C, temperate; D, cold; and E, polar. A second, mechanism-oriented grouping was used for the wet–arid contrasts. All B classes were arid; all E classes were polar; Af, Am and C/D classes with an f precipitation-season character were humid; all remaining recognised A, C and D classes were seasonally dry. This yielded 366 arid, 250 seasonally dry, 433 humid and 44 polar geocoded mines. The 78 unlocated mines were classified as unknown.

A subtype shift for mine $i$ in comparison $r\to k$ was

$Y_{i,r\to k}^{\mathrm{subtype}}=\mathbb{I}(C_{i,k}^{\mathrm{subtype}}\neq C_{i,r}^{\mathrm{subtype}})$	(S12)

The major-group indicator was defined analogously. Mine-count shift shares were arithmetic means of the binary indicators. An exploratory, coal-dominated tonnage-weighted subtype share was

$S_{r\to k}^{(w)}=\frac{\sum_{i}w_{i}Y_{i,r\to k}^{\mathrm{subtype}}}{\sum_{i}w_{i}}$	(S13)

Entry to and exit from the arid major group were also computed but were not used as standalone inferential endpoints in the main figures.

## Supplementary Method S6. Spatial-block bootstrap and descriptive uncertainty

Mines linked to the same or neighbouring climate cells are not statistically independent. We defined 1° spatial blocks by flooring the latitude and longitude of the sampled current-period 0.5° climate-cell centre. The 1,093 geocoded mines occupied 627 blocks. For each bootstrap replicate, the observed number of blocks was sampled with replacement, and every mine within a sampled block was carried together into the replicate. The estimate was recalculated on the concatenated records. The 2.5th and 97.5th percentiles of the 1,000 bootstrap estimates form the reported 95% interval. This procedure was used for mine-count and exploratory tonnage-weighted boundary-shift shares, medians of continuous climate changes within baseline hydroclimate regimes, and standardized open-pit versus underground differences. Quartiles reported for continuous metrics are empirical mine-level quartiles; the bootstrap interval quantifies uncertainty in the median under block resampling.

## Supplementary Method S7. Mine-level regression and method contrasts

The mine-level logistic model was restricted to the six prespecified commodities, the open-pit, underground and mixed method categories, and records with a recognised current major climate group. The final model contained 633 mines in 374 spatial blocks, including 258 projected subtype shifts under SSP5–8.5 for 2071–2099. Coal, open pit and arid climate were the reference categories. Absolute latitude was standardized using the analytical-sample mean of 31.105° and population standard deviation of 15.966°. The model was

$\mathrm{logit}\{\Pr(Y_{i}=1)\}=\beta_{0}+\boldsymbol{\beta}_{c}^{T}\mathbf{C}_{i}+\boldsymbol{\beta}_{m}^{T}\mathbf{M}_{i}+\boldsymbol{\beta}_{g}^{T}\mathbf{G}_{i}+\beta_{\ell}z(\left| \mathrm{latitude}_{i} \right|),$	(S14)

where $Y_{i}$ indicates a Köppen–Geiger subtype shift from 1991–2020 to SSP5–8.5 in 2071–2099, $\mathbf{C}_{i}$ is commodity, $\mathbf{M}_{i}$ is mining method, and $\mathbf{G}_{i}$ is the current major climate group. Standard errors were cluster-robust by 1° spatial block. Odds ratios and Wald 95% confidence intervals are reported. The Benjamini–Hochberg procedure [6] was applied to one prespecified family comprising the five non-reference commodity terms and two non-reference mining-method terms. Baseline climate-group and latitude terms were adjustment variables and were not included in that seven-term family.

The large adjusted odds ratio for the cold baseline group reflects strong dependence between a current climate category and whether its future subtype changes. It should not be read as a causal risk ratio or an operational effect. Commodity is likewise an incomplete proxy for orebody, host-rock and regional geological setting.

For a complementary method contrast, each projected SSP5–8.5 late-century metric was standardized across the combined geocoded open-pit and underground sample. The reported difference was

$D_{x}=\overline{z(x)}_{\mathrm{underground}}-\overline{z(x)}_{open pit},$	(S15)

where positive values indicate a larger standardized value among underground mines. These comparisons included 379 open-pit and 209 underground mines in 369 blocks. Their 95% intervals used the same 1° block-bootstrap procedure. These are unadjusted location contrasts, not within-geology comparisons.

## Supplementary Method S8. Tailings-facility disclosures

The facility analysis used the supplementary spreadsheet published with Franks et al. [3] (source-file title: “Supplementary Information 2”; XLSX), comprising 1,743 disclosed facilities. Yes/no strings were standardized case-insensitively while their source columns were retained. The relevant source question asks whether the facility has been, or is planned to be, assessed against more frequent extreme weather under climate change. A reported “No” was encoded as one, “Yes” as zero, and missing or unrecognised responses remained missing. The resulting assessment-response endpoint was available for 1,717 facilities: 220 reported “No” and 1,497 reported “Yes”. It is a disclosure response, not a safety classification. A similarly cleaned history-of-stability-concerns variable was retained during processing but was not the outcome of the reported multivariable model.

Height and current storage were converted to numeric values. Annual precipitation was converted from the disclosed AnnPrec field, and non-finite or out-of-range values outside 0–10,000 mm were set to missing. Status was grouped as active, inactive/closed or other. The dataset’s supplied raise categories were retained. The regression was restricted to downstream, upstream, centreline and hybrid facilities with complete outcome, non-negative height and storage, and valid annual precipitation. The complete-case model contained 1,262 facilities, 148 reported “No” assessment responses, and 239 owner clusters.

Height and storage were transformed as $\log(1+x)$ and then standardized; annual precipitation was standardized without log transformation. Within the model sample, the mean and population standard deviation were 3.298 and 0.851 for log-transformed height, 15.108 and 2.879 for log-transformed storage, and 862.31 and 577.13 mm for annual precipitation. The model was

$\mathrm{logit}\{\Pr(L_{j}=1)\}=\alpha_{0}+\boldsymbol{\alpha}_{r}^{T}\mathbf{R}_{j}+\alpha_{h}z\{\log(1+H_{j})\}+\alpha_{v}z\{\log(1+V_{j})\}+\alpha_{p}z(P_{j})+\boldsymbol{\alpha}_{s}^{T}\mathbf{S}_{j},$	(S16)

where $L_{j}$ denotes a reported “No” to the climate/extreme-weather assessment question, $\mathbf{R}_{j}$ is raise category, $H_{j}$ is height, $V_{j}$ is storage, $P_{j}$ is annual precipitation, and $\mathbf{S}_{j}$ is status. Downstream and active were the reference categories. Standard errors were cluster-robust by owner. Two records with missing owner were assigned distinct singleton cluster labels. Benjamini–Hochberg adjustment was applied to all eight non-intercept terms. The model quantifies patterns in self-reported answers; it is not an independent safety assessment.

## Supplementary Method S9. Tailings-failure events, multi-label causes and conditional consequences

The incident analysis used version 2 of the Rana et al. database [4]. The table contained 303 tailings-storage-facility failures from 1965 through 2020. Numeric fields were cleaned by removing non-breaking spaces, normalizing the Unicode minus sign and coercing unparseable values to missing. Coordinates were available for 258 events. Failure cause was available for 229 events.

An event was marked weather-involved when its multi-label cause string contained “Weather hazards”. Five reported cause tokens were retained: weather hazards, structural drainage deficiency, embankment deficiency, unstable foundation and seismic activity. Events with no recognised token were assigned to a residual “Unreported” group. For event $e$, with set of recognised labels $\mathcal{C}_{e}$, the fractional contribution of cause $c$ was

$f_{e,c}=\frac{\mathbb{I}(c\in\mathcal{C}_{e})}{\left| \mathcal{C}_{e} \right|}$	(S17)

Thus, each event contributed a total weight of one across causes. There were 261 single-label, 34 two-label and 8 three-label events, producing 353 event–cause rows whose fractional weights summed to 303. Decade summaries use these fractional counts and should not be interpreted as mutually exclusive primary causes.

Weather involvement was present in 64 of 229 events with a reported cause, or 27.95%. Consequence comparisons were conditional on a reported failure and, where applicable, a reported positive release volume. The release-volume empirical cumulative distributions included 33 weather-involved and 74 other known-cause events. Fatal-event proportions used all 64 weather-involved and 165 other known-cause events; 95% intervals were Wilson score intervals. The current version-2 table yields 2,024 deaths when the numeric death column is summed, whereas the associated article reports 2,023. We report the value recomputed from the frozen table and retain the discrepancy as a version-specific provenance note.

The Franks facility disclosures and Rana failures were not record-linked. Dividing the number of Rana events by the number of Franks disclosures would combine different populations, periods and selection mechanisms; no failure rate was therefore calculated.

## Supplementary Method S10. Missing data, multiplicity and interpretation

Analyses used available cases for each endpoint. The 78 unlocated Fineprint records remain in inventory and missingness summaries but do not enter spatial or climate analyses. Unknown mining method remains visible in descriptive panels and is excluded from models that require a method contrast. Tailings outcomes and covariates were not imputed. Missing failure causes were not reassigned to weather or non-weather groups.

No null-hypothesis test was applied to descriptive climate medians or shift proportions; emphasis is placed on effect estimates and spatial-block bootstrap intervals. Logistic-model $p$ values are two-sided Wald values. Benjamini–Hochberg adjusted $q$ values are reported only for the prespecified term families described above. An interval crossing its null value was treated as imprecise, not as proof of no association.

## Supplementary Method S11. Sensitivity contrasts performed

Robustness was assessed through contrasts that were available within the frozen data design. Climate-boundary change was reported at both the 30-subtype and five-major-group resolutions; the latter is a more conservative definition of crossing. Mine-count estimates were compared with an exploratory, coal-dominated 2015–2020 tonnage-weighted diagnostic. Mid-century and late-century windows were assessed under both SSP2–4.5 and SSP5–8.5, and the historical 1901–1930 to 1991–2020 contrast provided a non-scenario benchmark. Continuous precipitation metrics were examined separately from discrete climate-class shifts. Mining method was evaluated both as an adjusted regression term and as an unadjusted, spatially bootstrapped standardized contrast.

For tailings, raw facility distributions were shown alongside an owner-clustered adjusted model. Event causes were summarized with fractional multi-label counting rather than forcing one primary cause. Consequences were separated into fatal-event proportions and positive-volume distributions, with endpoint-specific sample sizes. No alternative climate product, field-monitoring dataset, geological map or causal identification design was analysed; these remain priorities for validation rather than completed sensitivity analyses.

## Supplementary Method S12. Software and reproducibility

The analytical environment used Python 3.12.14, NumPy 2.3.5, pandas 3.0.1, SciPy 1.18.1, statsmodels 0.15.0, netCDF4 1.7.4, Matplotlib 3.11.1 and seaborn 0.13.2. The analysis code preserves raw identifiers during processing and implements the reported joins, exclusions, summaries and figures. Natural Earth 1:110m country boundaries were used only as a public-domain cartographic backdrop and did not enter any calculation.

## Supplementary Results

## Supplementary Results S1. Represented mine inventory and hydroclimate coverage

The Fineprint inventory contained 1,171 top-level mines in 63 countries, of which 1,093 could be linked to all analysed climate normals and occupied 627 one-degree spatial blocks. The 78 unlocated records account for the identical 6.66% missingness in coordinates, climate class and continuous climate metrics. A positive 2015–2020 production weight was available for 658 mines, including 641 with a valid climate block; those 641 mines occupied 393 blocks. The six focal commodities contained 968 records. Of these, 657 also had an analysed mining method and 633 additionally had a recognised current major climate group, yielding the logistic-model sample in 374 blocks. The method contrast used 379 geocoded open-pit mines and 209 geocoded underground mines in 369 blocks.

The represented inventory comprised 446 coal, 197 gold, 135 copper, 109 iron, 41 nickel and 40 zinc mines, with 203 records assigned to other supplied commodity labels. Recorded mining method comprised 402 open-pit, 221 underground, 142 mixed and 406 unknown records. Among geocoded mines, the current major-group distribution was 366 arid, 252 temperate, 244 cold, 187 tropical and 44 polar. The mechanism-oriented hydroclimate grouping contained 433 humid, 366 arid, 250 seasonally dry and 44 polar records. The distinct distributions across commodities are consistent with geological and historical siting constraints, but the commodity labels do not identify host rock, deposit type, structural setting or mine-scale hydraulic conductivity.

## Supplementary Results S2. Boundary shifts and wet–arid divergence

The estimated mine-count share assigned to a different Köppen–Geiger subtype was 16.19% (spatial-block bootstrap 95% interval, 12.95–19.55%) between 1901–1930 and 1991–2020. Relative to 1991–2020, the subtype-shift share was 19.21% (15.94–23.25%) under SSP2–4.5 and 29.55% (25.00–34.07%) under SSP5–8.5 for 2041–2070, rising to 29.83% (24.98–34.99%) and 42.54% (36.81–47.49%), respectively, for 2071–2099. The corresponding major-group shares were 3.84% (2.65–5.19%) for the historical comparison, 8.78% (5.99–11.84%) and 11.71% (8.53–15.28%) at mid-century, and 11.71% (8.57–15.22%) and 16.93% (13.29–20.50%) by late century. Each mine-count estimate used 1,093 mines in 627 blocks. The exploratory late-century SSP5–8.5 tonnage-weighted subtype share was 48.57% (34.29–63.08%), but 95.75% of its summed weight came from coal. Its broad interval and cross-commodity unit dependence preclude interpretation as a global production-weighted exposure estimate.

Under late-century SSP5–8.5, current cold locations shifted mainly towards temperate ($n=42$) or arid ($n=24$) classes, whereas current temperate locations shifted towards tropical ($n=45$) or arid ($n=44$) classes. Of the 44 current polar locations, 24 crossed a major-group boundary: 3 shifted to arid, 7 to cold, and 14 to temperate conditions; the other 20 remained polar.

Under SSP5–8.5 in 2071–2099, baseline arid mine locations had a median annual precipitation change of −3.05% (−3.94 to −2.09%), wettest-quarter change of −1.51% (−2.20 to −0.63%), driest-quarter change of −1.84 mm (−2.50 to −0.88 mm), and seasonality-index change of +0.0141 (+0.0081 to +0.0201). Baseline humid locations instead had median annual precipitation change of +6.51% (+5.31 to +7.86%), wettest-quarter change of +3.13% (+1.69 to +5.00%), driest-quarter change of +9.00 mm (+7.09 to +11.25 mm), and seasonality-index change of −0.0064 (−0.0183 to +0.0005). Both groups warmed strongly on the common January–November basis. These are changes in regional climate normals, not observed mine-water balances.

## Supplementary Results S3. Commodity and mining-method strata

Commodity-level medians also differed: annual precipitation changed by +4.8% at coal and +7.4% at zinc locations, but by −0.5% at gold and −2.2% at iron locations. Subtype-shift shares ranged from 34.2% for gold to 55.0% for nickel, while all six groups warmed by 3.9–4.3 °C on the common January–November basis.

In the adjusted mine model, copper and iron records had higher odds of a late-century SSP5–8.5 subtype shift than coal records: adjusted odds ratio 3.77 (95% confidence interval, 1.70–8.36; $q = 0.0038$) for copper and 4.16 (1.80–9.62; $q = 0.0038$) for iron. The corresponding estimates were 1.21 (0.52–2.82; $q = 0.901$) for gold, 0.94 (0.18–4.85; $q = 0.941$) for nickel and 0.47 (0.12–1.88; $q = 0.674$) for zinc. Relative to open-pit records, the adjusted odds ratio was 1.10 (0.59–2.06; $q = 0.901$) for mixed records and 0.87 (0.43–1.77; $q = 0.901$) for underground records. These coefficients describe where commodity groups and methods are located relative to modelled climate boundaries; they do not demonstrate commodity-specific material sensitivity.

The unadjusted standardized method contrast showed larger projected January–November warming, annual precipitation change and wettest-quarter precipitation change at underground-mine locations than at open-pit locations. Standardized differences were 0.329 (0.138–0.531), 0.357 (0.136–0.561) and 0.227 (0.026–0.443), respectively. The standardized differences were −0.134 (−0.338 to 0.081) for seasonality and 0.215 (−0.0004 to 0.438) for subtype shift, so both intervals included zero. Because these comparisons do not match mines on geology or region, they are exposure-stratification results rather than evidence that mining method causes the difference.

## Supplementary Results S4. Tailings disclosures and reported failures

Of 1,743 disclosed facilities, 1,721 had usable numeric coordinates and 1,717 had a usable climate/extreme-weather assessment response; 220 of those 1,717 facilities (12.81%) reported “No”. Successive complete-case restrictions retained 1,647 records with non-negative height, 1,613 with non-negative storage, 1,583 with valid annual precipitation and 1,262 in the four analysed raise categories. The adjusted model therefore contained 1,262 facilities, 148 “No” responses and 239 owner clusters. All eight non-intercept confidence intervals crossed an odds ratio of one, and every adjusted $q$ value exceeded 0.72. The data therefore do not support ranking raise categories or facility attributes by this self-reported endpoint.

The failure database contained 303 reported events, including 258 with numeric coordinates and 229 with a reported cause. Among the known-cause events, 64 (27.95%) included weather hazards and 165 had other reported causes. Weather-involved events had 11 fatal events among 64 reports (17.2%; Wilson 95% confidence interval, 9.9–28.2%), compared with 16 among 165 other known-cause reports (9.7%; 6.1–15.2%). The positive-release-volume comparison contained 107 known-cause events: the median was 150,000 m³ (interquartile range, 15,000–600,000 m³; $n = 33$) for weather-involved events and 195,000 m³ (56,250–1,297,500 m³; $n = 74$) for other known causes. These overlapping, incident-conditional distributions do not establish that weather involvement increases event severity.

# Supplementary Limitations

The mine database is a documented global compilation, not a probability sample of all mines. Large operations, public companies and some commodities are better represented than small, informal or poorly disclosed operations. Coordinates are points and cannot resolve the spatial extent of pits, underground workings, waste facilities or hydrological catchments. The climate products describe regional 30-year normals; they do not measure mine-site rainfall intensity, evaporation, recharge, pore pressure, dewatering demand, ventilation load or operational disruption. Discrete Köppen boundaries can change when a continuous climate variable crosses a threshold, so major-group results and continuous metrics are necessary context for subtype shifts.

Mining method is inferred from a text field and is missing or unclassified for more than one-third of records. Commodity captures only part of geological heterogeneity and cannot substitute for deposit type, host lithology, structural fabric, alteration, depth, permeability or mine design. The logistic models are cross-sectional scenario models and can be affected by sparse cells, residual spatial structure and the deterministic relationship between baseline climate class and future class transition. Their odds ratios are not causal effects.

The exploratory tonnage-weighted estimate combines physical tonnes across commodities with very different mass scales and economic meanings. Coal contributes 95.75% of the summed weight and iron 3.70%, so the result is effectively coal-dominated. It is retained only as a diagnostic of weight concentration and must not be presented as global production-weighted exposure or economic output at risk.

The Franks dataset reflects a disclosure initiative and self-reported statements. A reported “No” to the assessment question is not an observed failure probability, and a reported “Yes” is not an independent engineering audit. The complete-case model excludes less common raise categories and records with missing numerical fields. Owner-clustered intervals address within-owner dependence but not selection into disclosure or unmeasured design and governance differences.

The Rana database compiles reported failures and lacks a complete global facility-time denominator. Early, small or poorly documented events may be under-represented. Failure causes are retrospective, sometimes missing and often multi-label. The fractional-counting approach preserves one unit of total weight per event but cannot identify a primary trigger. Release-volume and fatality comparisons are conditional on a reported incident and endpoint availability. Franks facilities and Rana incidents must remain analytically separate. Together, these constraints support the Perspective’s central proposition as a testable systems framework, while reserving causal attribution for mine-specific monitoring and prospective validation.

# Supplementary References

1. Jasansky, S., Lieber, M., Giljum, S. & Maus, V. An open database on global coal and metal mine production. *Scientific Data* **10**, 52 (2023). https://doi.org/10.1038/s41597-023-01965-y. Dataset: https://doi.org/10.5281/zenodo.7369478.

2. Beck, H. E. et al. High-resolution (1 km) Köppen-Geiger maps for 1901–2099 based on constrained CMIP6 projections. *Scientific Data* **10**, 724 (2023). https://doi.org/10.1038/s41597-023-02549-6. Collection: https://doi.org/10.6084/m9.figshare.c.6395666.v1.

3. Franks, D. M. et al. Tailings facility disclosures reveal stability risks. *Scientific Reports* **11**, 5353 (2021). https://doi.org/10.1038/s41598-021-84897-0.

4. Rana, N. M. et al. Global magnitude-frequency statistics of the failures and impacts of large water-retention dams and mine tailings impoundments. *Earth-Science Reviews* **232**, 104144 (2022). https://doi.org/10.1016/j.earscirev.2022.104144. Dataset: https://doi.org/10.5683/SP3/K7SANT.

5. Walsh, R. P. D. & Lawler, D. M. Rainfall seasonality: description, spatial patterns and change through time. *Weather* **36**, 201–208 (1981). https://doi.org/10.1002/j.1477-8696.1981.tb05400.x.

6. Benjamini, Y. & Hochberg, Y. Controlling the false discovery rate: a practical and powerful approach to multiple testing. *Journal of the Royal Statistical Society: Series B (Methodological)* **57**, 289–300 (1995). https://doi.org/10.1111/j.2517-6161.1995.tb02031.x.
